# Assessing the Efficacy and Immunogenicity of Anticaries Vaccine—A Systematic Review and Meta‐Analysis

**DOI:** 10.1002/iid3.70253

**Published:** 2025-09-05

**Authors:** Gunjan Kumar, Payal Dash, Smruti Bhusan Nanda, Mohammad Fareed, Mohmed Isaqali Karobari

**Affiliations:** ^1^ Department of Public Health Dentistry, Kalinga Institute of Dental Sciences (KIDS) Kalinga Institute of Industrial Technology (KIIT) Deemed to Be University Bhubaneswar India; ^2^ Mamata Orthodontic Clinic Saheed Nagar Bhubaneswar India; ^3^ Department of Basic Medical Sciences, College of Medicine AlMaarefa University Riyadh Kingdom of Saudi Arabia; ^4^ Department of Conservative Dentistry and Endodontics, Saveetha Dental College and Hospitals, Saveetha Institute of Medical and Technical Sciences Saveetha University Chennai Tamil Nadu India; ^5^ Department of Conservative Dentistry and Endodontics, Faculty of Dentistry University of Puthisastra Phnom Penh Cambodia

**Keywords:** dental caries, dental caries vaccines, immunogenicity, vaccine efficacy

## Abstract

**Objectives:**

The current systematic review and meta‐analysis aims to assess the safety and immunogenicity of the anticaries vaccines currently available.

**Materials and Methods:**

Electronic searches of PubMed, Scopus, Web of Science and Science Direct were performed. Studies assessing the safety and effectiveness of the anticaries vaccination, including the dose, plasmids, serum IgG and IgA levels after immunization, were included. Studies in which animals were used for anticaries vaccine were also included. Combining more than one trial was done to estimate the pooled intervention effect using the meta‐analysis when studies examined the same intervention and outcomes with comparable methods in similar populations. The continuous data was pooled using the inverse variance method, and dichotomous data using the Mantel‐Haenszel method.

**Results:**

Out of 4701 records, only 17 publications met the eligibility criteria. Although all studies were assessed as having an overall low risk of bias, certain domains (D3, D5, and D7) exhibited a high risk across most studies. The pooled RR, derived using a random‐effects model, was 0.53 (95% CI: 0.46–0.62), indicating a statistically significant 47% reduction in risk across studies.

**Conclusions:**

There is excellent potential for dental caries vaccines to transform oral health in the future. Various vaccines, such as Killed Formalin‐treated Donor strain 2 ‐ recombinant Protein Antigen c and anti‐CAT‐SYIIgY antibodies, have demonstrated prophylactic effects against Streptococcus mutans colonization on teeth. These interventions exhibit a sustained reduction in S. mutans colonization, which may contribute to the prevention of dental caries. A vaccination of this kind would significantly lower tooth decay prevalence and the financial and health costs that go along with it.

## Graphical abstract

Although there isn't currently a vaccination that is widely used, advances in the field and further study indicate that one could be developed in the near future to prevent dental caries. •A vaccination of this kind would significantly lower tooth decay prevalence and the financial and health costs that go along with it.•Additional study, clinical studies, and regulatory permissions are necessary for the development and successful deployment. The search for dental caries vaccines is an appealing path for future developments in healthcare because of the possible influence on oral health around the world.

## 1 Introduction

Dental caries is one of the most common disorders affecting people, causing severe health concerns as well as a significant financial burden, according to the World Health Organization (WHO) [1]. Epidemiologic research has also revealed that child caries rates have lately climbed in certain industrialized nations following many years of declining rates despite enhanced access to public and private health measures [2]. In addition, many developing countries' populace suffer dental caries due to inadequate social infrastructure or poverty [3]. Vaccination has long been recognized as a powerful tool in the prevention of infectious diseases. However, developing a vaccine against dental caries presents unique challenges due to its multifactorial etiology and the complex ecology of the oral cavity.

While Streptococcus mutans (S. mutans) has historically been associated with dental caries, modern research suggests that caries development is a multifactorial process, and S. mutans is no longer considered the sole causative organism [4, 5]. Cavities can result from Streptococcus mutans and other acidogenic bacteria producing acids that demineralize the subsurface enamel following plaque biofilm formation [6]. Research into anticaries vaccines began in the early 1970s. In 1972, reports from England described successful preclinical trials in animals, with preparations underway for human testing. These early efforts laid the groundwork for subsequent exploration of immunological targets and delivery systems. Over the decades, vaccine development has focused on critical virulence factors of *S. mutans*, including glucosyltransferases (GTFs), antigen I/II (PAc), and glucan‐binding proteins (GBPs), which play essential roles in colonization and cariogenicity. Recombinant vaccines based on these antigens have demonstrated the capacity to elicit robust immune responses in preclinical models [7].

Developing an effective vaccine against dental caries is inherently complex due to the biological characteristics of Streptococcus mutans and the anatomical challenges of the oral cavity. S. mutans displays considerable genetic and antigenic variability, which complicates the creation of a universally protective vaccine. Additionally, the oral environment is highly dynamic—constantly influenced by saliva, food, and pH changes—which makes local vaccine delivery particularly difficult. Mucosal routes such as oral and intranasal administration are attractive for targeting S. mutans at the point of colonization, but they pose significant obstacles. These include antigen degradation by digestive enzymes, poor mucosal absorption, and the need for potent adjuvants to trigger a sufficient immune response. Recent innovations—such as mucoadhesive systems, biodegradable nanoparticles, and mucosal adjuvants—are being explored to overcome these barriers and enhance both antigen stability and immune activation.

Emerging platforms such as DNA and peptide‐based vaccines offer additional promise by enabling precise antigen design and long‐lasting immune responses. DNA vaccines, for instance, can induce both humoral and cellular immunity by promoting endogenous antigen production. Peptide vaccines, meanwhile, provide targeted immunogenic regions of bacterial proteins and are easier to synthesize and modify.

Currently, widely used alternatives include fluoride‐based treatments, dental sealants, and daily oral care practices. Fluoride—delivered through toothpaste, water fluoridation, or professional applications—strengthens enamel and helps prevent demineralization. Dental sealants protect the deep grooves of teeth by preventing plaque and food accumulation, while regular brushing, flossing, and dietary control remain essential components of caries prevention. However, these measures rely heavily on individual adherence and regular access to dental services.

Despite substantial progress, no anticaries vaccine has yet received regulatory approval. Key barriers include the genetic variability of *S. mutans*, the dynamic oral environment, and challenges in inducing effective and sustained mucosal immunity. Nevertheless, vaccines remain an attractive complementary strategy to existing preventive measures such as fluoride therapy, dental sealants, and personal oral hygiene—particularly for populations with limited access to dental care. Concerns also remain regarding the safety of anticaries vaccines. Notably, some antigens used in vaccine formulations such as Protein Antigen c (PAc) share structural similarities with human cardiac tissue proteins. This raises the possibility of cross‐reactive immune responses, potentially leading to cardiac autoimmunity. Although no definitive adverse cardiac effects have been confirmed in animal studies, this theoretical risk underscores the importance of careful antigen selection, epitope mapping, and long‐term safety monitoring in the development of caries vaccines. Hence, this review aims to assess the efficacy and immunogenicity of the anticaries vaccine.

## 2 Materials and Methods

### 2.1 Protocol Registration

The systematic literature search was conducted according to the Preferred Reporting Items for Systematic Reviews and Meta‐Analyses statement guidelines (Supporting Information S1: File S1). This review was registered with the International Prospective Register of Systematic Reviews. (Registration Number CRD42023453019).

The Population, Intervention, Comparison, Outcome question defined the focussed question such as “What is efficacy and immunogenicity of the anticaries vaccine?” where,

o **Population**: Animal models (rats, mice, rabbits); transgenic plants.
o **Intervention**: Anticaries vaccines (recombinant, DNA‐based, plant‐based).
o **Comparison**: Placebo, fluoride, chlorhexidine, no intervention.
o **Outcomes**: Reduction in *S. mutans* colonization, serum/salivary IgA or serum IgG levels.
o **Study Type**: Preclinical in vivo studies.

### 2.2 Inclusion Criteria

This systematic review included peer‐reviewed articles that have been published in English language and were indexed in reputable databases such as PubMed, Scopus, Science direct and Web of Science. The main objective of the included studies focussed on assessing the effect of anticaries vaccines, including recombinant vaccines, plant‐based vaccines, or DNA vaccines, targeting *Streptococcus mutans*. Eligible studies primarily involved animal models (nonhuman primates, rodents) or plant‐based systems (transgenic plants) that evaluated the efficacy of vaccines in reducing *S. mutans* colonization or improving immunogenicity.

### 2.3 Exclusion Criteria

Manuscripts that had not been published, such as abstracts, conference proceedings, dissertations, and other forms of gray literature, were excluded from the review. Studies that did not focus on the effect of anticaries vaccines targeting Streptococcus mutans were also excluded. Articles in languages other than English were not considered. Additionally, case reports, case series, and review articles that did not present original research or preclinical data were excluded.

### 2.4 Search Strategy

Two authors (G.K., P.D.) performed an exhaustive literature search to assess the anticaries vaccination′s safety and efficacy, including the dose, plasmids, serum IgG and IgA levels after immunization. Online electronic databases such as PubMed, Scopus, Web of Science and Science Direct upto August 2025 were used to search data. The search results were downloaded to a bibliographic database to facilitate the removal of duplicate entries. Authors were contacted for any unpublished studies.

Moreover, supplementary citations that were acknowledged through the lists of selected references and bibliographic linkages were also integrated into this review. Medical Subject Headings (MeSH) terms, keywords, and other accessible terms combined with Boolean operators (OR, AND) were used to search articles. Identical keywords were used for all the search platforms following the syntax rules of each database. A detailed search is given in Table 1. The complete search strategy for databases including Boolean operators, search terms, filters, and date ranges, is provided in Supporting Information S1: Table S1.

**Table 1:** Sources of information and search strategies.

Database	Search Strategy (Keywords, MeSH, Boolean Operators)	Number of results
PubMed‐MEDLINE	(((“efficacies”[All Fields] OR “efficacious”[All Fields] OR “efficaciously”[All Fields] OR “efficaciousness”[All Fields] OR “efficacy”[All Fields]) AND (“dental caries”[MeSH Terms] OR (“dental”[All Fields] AND “caries”[All Fields]) OR “dental caries”[All Fields])) OR ((“antib technol j”[Journal] OR “anti”[All Fields]) AND (“carie”[All Fields] OR “dental caries”[MeSH Terms] OR (“dental”[All Fields] AND “caries”[All Fields]) OR “dental caries”[All Fields] OR “caries”[All Fields]))) AND (“vaccin”[Supplementary Concept] OR “vaccin”[All Fields] OR “vaccination”[MeSH Terms] OR “vaccination”[All Fields] OR “vaccinable”[All Fields] OR “vaccinal”[All Fields] OR “vaccinate”[All Fields] OR “vaccinated”[All Fields] OR “vaccinates”[All Fields] OR “vaccinating”[All Fields] OR “vaccinations”[All Fields] OR “vaccination s”[All Fields] OR “vaccinator”[All Fields] OR “vaccinators”[All Fields] OR “vaccine s”[All Fields] OR “vaccined”[All Fields] OR “vaccines”[MeSH Terms] OR “vaccines”[All Fields] OR “vaccine”[All Fields] OR “vaccins”[All Fields])	162
Scopus	(((ALL(efficacies) OR ALL(efficacious) OR ALL(efficaciously) OR ALL(efficaciousness) OR ALL(efficacy)) AND (INDEXTERMS(“dental caries”) OR (ALL(dental) AND ALL(caries)) OR ALL(“dental caries”))) OR ((SRCTITLE(“antib technol j”) OR ALL(anti)) AND (ALL(carie) OR INDEXTERMS(“dental caries”) OR (ALL(dental) AND ALL(caries)) OR ALL(“dental caries”) OR ALL(caries)))) AND (CHEM(term) OR ALL(vaccin) OR INDEXTERMS(vaccination) OR ALL(vaccination) OR ALL(vaccinable) OR ALL(vaccinal) OR ALL(vaccinate) OR ALL(vaccinated) OR ALL(vaccinates) OR ALL(vaccinating) OR ALL(vaccinations) OR ALL(“vaccination s”) OR ALL(vaccinator) OR ALL(vaccinators) OR ALL(“vaccine s”) OR ALL(vaccined) OR INDEXTERMS(vaccines) OR ALL(vaccines) OR ALL(vaccine) OR ALL(vaccins))	3790
Web of Science	(((efficacies OR efficacious OR efficaciously OR efficaciousness OR efficacy) AND (“dental caries” OR (dental AND caries) OR “dental caries”)) OR ((“antib technol j” OR anti) AND (carie OR “dental caries” OR (dental AND caries) OR “dental caries” OR caries))) AND (vaccin OR vaccin OR vaccination OR vaccination OR vaccinable OR vaccinal OR vaccinate OR vaccinated OR vaccinates OR vaccinating OR vaccinations OR “vaccination s” OR vaccinator OR vaccinators OR “vaccine s” OR vaccined OR vaccines OR vaccines OR vaccine OR vaccins)	494
Science direct	(“dental caries” AND (vaccine OR vaccination) AND efficacy)	255
Total		4701

### 2.5 Screening and Selection

The study′s authors separately went through the literature search results to look for anticaries vaccines. The eligibility screening procedure is divided into two stages: the first screens the titles and abstracts of the obtained records, and the second screens the full‐text articles of the abstracts selected in the first stage. Discussions with the supervisor and subject matter expert were held to address any discrepancies arising from the conclusion. Two reviewers (G.K., P.D.) independently used the RAYYAN program to conduct a selection process to finish the first round. Discussions or the involvement of the third reviewer resolved disputes.

### 2.6 Data Extraction

Authors individually retrieved the data using a pre‐arranged data extraction form. From each included study, the following variables were extracted using a standardized data extraction form:

Study Characteristics: First author, journal name, year of publication, study location, total sample size, and target population or animal model.

Intervention Details: Type of anticaries agent (vaccine, adjuvant, or antibody), number of immunizations, and details of plasmids or constructs used.

Immunological Outcomes: Type of antibody response (salivary IgA, serum IgG), magnitude or levels of immune response reported.

Clinical/Microbiological Outcomes: Caries prevention efficacy metrics, including reduction in Streptococcus mutans colonization, CFU counts, and lesion scores.

To evaluate the consistency between reviewers, Cohen's kappa statistic was calculated. The kappa value was found to be 0.78, indicating substantial agreement. Discrepancies were resolved through discussion or consultation with a third reviewer.

### 2.7 Risk of Bias

Using the SYRCLE risk‐of‐bias tool, two blinded reviewers (G.K., P.D.) assessed the internal validity of the included studies. This tool, adapted from the Cochrane risk‐of‐bias framework, accounts for bias domains specific to animal intervention studies. This tool comprises 10 domains (D1–D10) that cover six categories of bias: selection bias, performance bias, detection bias, attrition bias, reporting bias, and other sources of bias. Specifically, D1 refers to sequence generation, D2 to baseline characteristics, and D3 to allocation concealment, all of which address selection bias. D4 covers random housing and D5 evaluates the blinding of caregivers or investigators, addressing performance bias. Detection bias is assessed through D6 (random outcome assessment) and D7 (blinding of outcome assessors). Attrition bias is examined under D8, which pertains to incomplete outcome data, while D9 addresses reporting bias through assessment of selective outcome reporting. Finally, D10 accounts for any other potential sources of bias. Each domain was independently rated as having a low, high, or unclear risk of bias by two reviewers, with any disagreements resolved through discussion and consensus.

To ensure methodological rigor, studies were included only if they used in vivo animal models with appropriate controls and reported relevant outcome measures related to caries reduction or immunogenicity. Studies were excluded if they lacked a control group, did not report quantitative outcomes, were reviews or conference abstracts, or were not published in English.

A summary of the risk‐of‐bias assessment is presented in Table 4. Most studies showed unclear risk for allocation concealment and blinding of caregivers or outcome assessors due to incomplete reporting. Many of the included studies exhibited a high or unclear risk of bias across multiple domains. Although these studies were not excluded based on quality scores, the presence of bias may have affected the reliability of the reported outcomes. Due to the heterogeneity in methodologies and outcome measures, a formal sensitivity analysis could not be conducted; however, the risk of bias was considered when interpreting and synthesizing the overall evidence.

### 2.8 Data Analysis

The meta‐analysis was conducted using Review Manager (RevMan), version 4.2 for Windows (The Nordic Cochrane Centre, The Cochrane Collaboration, Copenhagen, Denmark). Studies were pooled when they examined similar interventions and outcomes in comparable animal populations. Continuous outcomes were combined using the inverse variance method, and dichotomous outcomes were analyzed using the Mantel‐Haenszel method.

Effect sizes for dichotomous outcomes were expressed as risk ratios (RRs) with 95% confidence intervals (CIs). For continuous outcomes, mean differences with 95% CIs were used. Where studies measured the same outcome using different scales or methods, standardized mean differences with 95% CIs were reported.

#### 2.8.1 Assessment of Heterogeneity and Model Selection

Statistical heterogeneity among studies was assessed using the Chi‐square (χ²) test and quantified using the *I*² statistic. *I*² values describe the proportion of total variation across studies that is due to heterogeneity rather than chance, ranging from 0% (no heterogeneity) to 100% (substantial heterogeneity). *I*² values above 50% or a χ² test p‐value less than 0.1 were considered indicative of significant heterogeneity.

In instances of low heterogeneity (*I*² ≤ 50%, *p* ≥ 0.1), a fixed‐effect model was applied, assuming that all studies estimate the same underlying effect. However, when heterogeneity was moderate to high (*I*² > 50% or *p* < 0.1), a random‐effects model was used. This model assumes variability in true effect sizes across studies and incorporates both within‐study and between‐study variance, thus providing a more conservative and generalizable estimate—especially appropriate in preclinical animal research where differences in species, vaccine formulations, administration routes, and measurement methods exist.

This meta‐analysis is limited by the exclusive inclusion of animal studies, which restricts the generalizability of findings to human populations. Methodological heterogeneity—such as differences in species, vaccine protocols, and outcome assessments—contributed to variability across studies. Additionally, incomplete reporting of key methodological details in several studies may have introduced bias. The potential for publication bias also exists, though it could not be reliably assessed due to the limited number of studies.

#### 2.8.2 Assessment of Reporting Bias

Potential publication bias was examined using funnel plots. Asymmetry in the plot may suggest the presence of reporting bias or small‐study effects. However, due to the limited number of studies in some comparisons, the ability to detect bias using this method may be constrained.

## 3 Results

### 3.1 Study Selection Results

**Figure 1:**
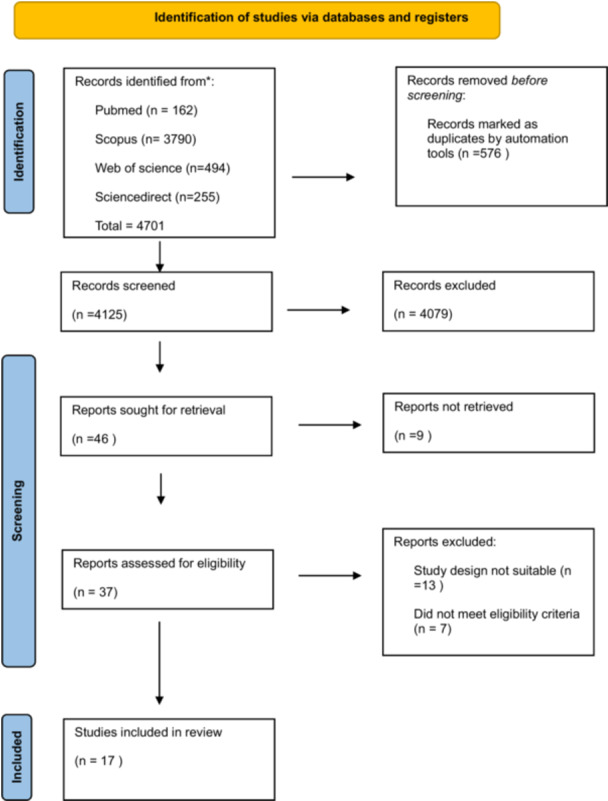
Flow chart summarizing the article selection process.

Through a systematic literature search across the databases, a total of 4701 articles were yielded. After removing 576 duplicates, 4125 articles were scrutinized based on title and abstract screening. For further full‐text evaluation, 37 articles were assessed for inclusion criteria, and finally, 17 articles were included for data extraction. (Figure 1).

### 3.2 Study Features

Table 2 summarizes the essential characteristics of the integrated articles. The included publications were published in a reputable journal listed in PubMed indexes. Acta pharmacologica sinica published the majority of the studies, vaccine published two, and the remaining studies were published once each in the following journals: Medical Hypotheses, Journal of Dentistry, Archives of Oral Biology, Brazilian Journal of Microbiology, Biotechnology and Applied Biochemistry, Immunobiology, Microbiology Immunity and Vaccines, Microbiology Spectrum and Vaccine.

**Table 2:** Characteristics of the included studies.

SI. no.	Author	Journal	Year of publication	Study location	Total sample	Target population
1	Yu Yb [8]	Microbiology Spectrum	2023	China	In vitro and in vivo	Female BALB/c mice aged 6 weeks
2	Zhang F [9]	Acta Pharmacologica Sinica	2007	China	96	Hamsters
3	Gisela De Souza Pereira [10]	Brazilian Journal Of Microbiology	2022	Brazil	5	BALB/c mic
4	Bai G [11]	Biotechnology And Applied Biochemistry.	2019	China	—	Tomato
5	Hao Jiang [12]	Immunobiology	2017	China		6week BALB/c mice
6	Yan‐Hong Yan [13]	Acta Pharmacologica Sinica	2016	China		6week BALB/c mice
7	Rong Jia [14]	Vaccine	2020	China	24	6‐week‐old female BALB/c mice
8	R. Bao [15]	Research Reports: Biological	2015	China	50	18‐d‐old female weanling Wistar rats
9	Milene Tavares Batista [16]	Vaccine	2017	USA	5	BALB/c, female, aged 6–8 weeks,
10	Sun, Jinghua [17]	Vaccine	2009	China	36	female BALB/c mice 8‐week‐old
11	Hongjiao l [18]	Nanomedicine	2016	China	40	female Sprague–Dawley rats (aged 21 days)
12	Liu, C [19]	Vaccine	2008	China	32	female Wistar rats
13	Xu Qa [20]	Caries Research	2005	China	36	female BALB/c mice
14	Liulin Chen [21]	Plosone	2013	China	50	6week BALB/c mice
15	Yang, Yp [22]	Acta Pharmacologica Sinica	2009	China	18	female Wistar rats
16	Yunxiaodu [23]	International Dental Journal	2024	China	48	42‐day‐old Sprague‐Dawley (SD) rats
17	Bowen Liu [24]	Human Vaccines & Immunotherapeutics	2024	China	36	Female Wistar rats

### 3.3 Study Characteristics

In this review, a total of seventeen published articles were included. China conducted the most significant number of investigations (15), followed by the USA (1) and São Paulo (1). The survey was distributed between 2005 and 2024. The survey by Zhang F et al. had the highest sample size, comprising 86 respondents. Of the seventeen investigations, nine focused on female BALB/c mice that were 6 weeks old; four surveys examined female Wistar rats; one examined tomatoes; one examined hamsters; and two examined female Sprague‐Dawley rats. (Table 3).

**Table 3:** Anticaries vaccine included in different studies.

SI. no.	Author	Vaccine/Adjuvant/Antibody	Number of immunizations	Plasmids	Antibody response (type and levels)	Antibody response and immunogenic proxies for efficacy
1	Yu Yb [8]	ZIF‐8 NPs	3 1st–2 weeks 2nd–10 weeks 3rd–12 weeks		High levels of serum IgG, IgG1, and IgG2a were observed at 5 weeks post‐immunization, with low levels noted at 2 weeks and elevated again at 10–12 weeks, indicating a biphasic humoral response.	In rats, immunization resulted in a significant antibody response, indicating effective immunogenicity.
2	Zhang F [9]	CTL4 fusion Anticaries DNA vaccine	Two doses	(pGJA–P/VAX1 and pGJA–P) pGLUA–P pCI pVAX1	Immunization with pGJA–P/VAX1 and pGJA–P resulted in significantly higher antibody titers compared to other plasmids, demonstrating strong immunogenicity.	Hamsters immunized with pGJA–P/VAX1 and pGJA–P exhibited noticeably fewer caries lesions, demonstrating strong protective efficacy.
3	Gisela De Souza Pereira [10]	LTK63	3		Administration of 5 µg rGlnH alone produced a low antibody response, whereas Coadministration with 5 µg LTK63 enhanced the response; however, no significant change was observed in IgA levels.	The formulation led to a significant reduction in caries caused by *Streptococcus mutans* NG8 strain, indicating functional antibody‐mediated protection.
4	Bai G [11]	fusion Anticaries DNA vaccine (PAcA‐ctxB)		pCAMBI‐35s and pBPC55	GUS gene expression was confirmed by blue coloration in transformed tomato plants, indicating successful antigen expression through transgenic delivery.	The expression of the GUS gene in tomato sprouts and shoots confirmed successful transgenic antigen delivery, validating the feasibility of plant‐based vaccines.
5	Hao Jiang [12]	S. typhimurium strain SL3261 and CHO cells	Fourdoses Day 0 Day 14 Day 28 Day 42	Recombinant SBR and GBR were expressed using pTriEx‐4‐SBR and pTriEx‐4‐GBR pCN‐SSIE and pCN‐SS/SG	Moderate serum IgG levels were recorded at 3 and 5 weeks post‐immunization with pNir‐SS/SG and pCN‐SS/SG, while a booster at 16 weeks with SL3261/pCN‐SS/SG induced a substantial increase in serum IgG.	A booster dose enhanced serum IgG levels, suggesting improved immunogenicity and potential for enhanced protection.
6	Yan‐Hong Yan [13]	DNA vaccine pCIA‐P	Fourdoses Day 0 Day 1 Week 2 and 4 (Booster dose)	COS‐7 cells with pCCL19/ GFP	Progressive increase in serum IgG levels was observed from weeks 4 to 14, particularly with coadministration of pCIA‐P and pCCL19/GFP; the response was further characterized by high IFN‐γ production.	Coadministration of pCIA‐P with an immune‐enhancing plasmid (pCCL19/GFP) resulted in better antibody responses, indicating improved immunity and adjuvant effectiveness.
7	Rong Jia [14]	pGLUAP + miR‐9 sponge pGLUA‐P + NC sponge Vector + miR‐9 sponge Vector + NC sponge	Booster dose ‐week 2	pGJA‐P/cDNA	The group receiving pGLUAP+miR‐9 showed elevated serum IgG at 2 weeks, which peaked at 4 weeks in the NC sponge group and declined by 50% at 6 weeks in the vector+miR‐9 group.	A significant difference in antibody levels at 8 weeks was observed between animals receiving pGLUAP+miR‐9 and those receiving the vector+miR‐9, highlighting the role of genetic modulation in enhancing immune response.
8	R. Bao [15]	Recombinant rPAc and fusion protein KF‐rPAc			Extremely high levels of IgA (415‐fold) and serum IgG (434‐fold) were observed in animals immunized with KF‐rPAc, indicating potent systemic and mucosal immune activation.	The KF‐rPAc vaccine showed superior efficacy, as reflected by strong humoral and mucosal immune responses.
9	Milene Tavares Batista [16]	P1 (aka AgI/II, PAc) adhesin (P139‐512) with LTK4R	Three doses		After the third dose, there was a marked increase in serum IgG and IgG2a, along with a decrease in the IL‐6/IFN‐γ ratio, suggesting a Th1‐biased immune response.	Immunization induced a robust Th1‐type immune response, evidenced by increased IgG2a levels and decreased IL‐6/IFN‐γ ratio, with subcutaneous delivery proving effective.
10	Sun, Jinghua [17]	pGJA‐P/VAX, (GLU fragment of the S. mutans gtfB gene and the A‐P fragment of the S. mutans pac gene)	Two doses (Day 0 and 14)	pGJGAC/VAX pGJGA‐5C/VAX	Elevated levels of serum anti‐PAc, GLU, and CAT IgG were noted after the second dose, indicating a robust antigen‐specific response.	Among the tested constructs, pGJGA‐5C/VAX elicited the strongest antibody response, indicating its superior protective potential.
11	Hongjiao l [18]	pVAX1‐wapA.	Four doses at 1 week interval	pVAX1	High serum IgG titers were recorded at both 5 and 8 weeks post‐immunization, indicating sustained antibody production over time.	The anti‐WapA vaccinated group showed enhanced protection, as seen in the reduction of caries lesions.
12	Liu, C [19]	pGJA‐P/VAX	Four doses at 1 week interval	pGJA‐P/VAX	Intramuscular immunization resulted in elevated systemic IgG, while intranasal administration elicited strong site‐specific IgA responses, suggesting effective mucosal and systemic immunogenicity.	The intranasally (i.n.) immunized group displayed fewer enamel and dentin lesions, confirming the effectiveness of mucosal immunization routes.
13	Xu Qa [20]	pGJA‐P	Two doses (Day 0 and 14)	pGJA‐P, pGLUA‐P and pCI	The pGJA–P group showed higher levels of both serum IgG and IgA compared to the pGLUA–P group, suggesting superior immunogenicity.	The pGJA‐P group demonstrated greater caries reduction efficacy compared to pGLUA‐P, affirming its superior protective potential.
14	Liulin Chen [21]	CS/DNA and AL/CS/DNA NPs	Naked DNA (i.n.), naked DNA (i.m.), CS/DNA (i.n.), AL/CS/DNA (i.n.) and PBS group (i.n.) two doses	pGJA‐P/VAX pcDNA3.0‐Rluc	High levels of IgA were recorded following administration of CS/DNA and AL/CS/DNA nanoparticles, with minimal cytotoxicity, supporting their potential for nasal mucosal delivery.	The nasal vaccine delivery using CS/DNA and AL/CS/DNA nanoparticles showed promising results, combining high IgA titers with minimal cytotoxicity, making it a viable mucosal delivery method.
15	Yang, Yp [22]	pGJA‐P/VAX	Group A‐pGJA‐P/VAX(G)‐chitosan complex Group B‐pGJA‐P/VAX(L)‐chitosan complex Group C‐pVAX1‐chitosan complex	pGJA‐P/VAX	Groups A and B exhibited marked increases in serum IgG and IgA against PAc and Glu, while Group C showed lower antibody titers, indicating differential immunogenicity across formulations.	Groups A and B, which received pGJA‐P/VAX‐chitosan complexes, exhibited caries reduction of 40.9% and 47.0% respectively, while Group C showed a lesser effect, confirming the importance of vaccine formulation.
16	Yunxiaodu [23]	CAT‐SYI fusion gene	Three doses (second and third dose at 2 week interval)	pET32a‐CAT‐SYI, pET32a‐GtfB, pET32a‐GbpB, andpET32a‐SpaP	Immunization with CAT‐SYI fusion gene resulted in high antibody titers (> 0.1 mg/mL), reflecting strong humoral response to the recombinant antigens.	Immunization with CAT‐SYI fusion gene constructs led to a reduction in caries scores ranging from 66.5% to 89%, as evaluated using Keyes' scoring system.
17	Bowen Liu [24]	KFD2‐rPAc	Three doses at 28 days interval	pET28a	Antibody levels were not reported for the KFD2‐rPAc group, though efficacy was evaluated via caries reduction scores.	Though antibody titers were not reported, the group immunized with KFD2‐rPAc demonstrated a significant reduction in enamel and dentin caries, achieving 83% inhibitory efficacy.

**Table 4:** Risk of bias assessment of included studies using SYRCLE tool.

Study	D1	D2	D3	D4	D5	D6	D7	D8	D9	D10	Overall
1	Low	Low	High	Low	High	Low	High	Low	Low	Low	Low
2	Low	Low	High	Low	High	Low	High	Low	Low	Low	Low
3	Low	Low	High	Low	High	Low	High	Low	Low	Low	Low
4	Low	Low	High	Low	High	Low	No information	Low	No information	No information	Low
5	Low	Low	Low	Low	No information	Low	High	Low	Low	Low	Low
6	Low	Low	High	Low	High	Low	High	Low	Low	Low	Low
7	Low	Low	Low	Low	High	Low	No information	Low	Low	Low	Low
8	Low	Low	High	Low	High	Low	High	Low	Low	Low	Low
9	Low	Low	High	Low	High	Low	High	Low	Low	Low	Low
10	Low	Low	High	Low	High	Low	High	Low	Low	Low	Low
11	Low	Low	High	Low	High	Low	High	Low	Low	Low	Low
12	Low	Low	High	Low	High	Low	High	Low	Low	Low	Low
13	Low	Low	High	Low	High	Low	High	Low	Low	Low	Low
14	Low	Low	High	Low	High	Low	High	Low	Low	Low	Low
15	Low	Low	High	Low	High	Low	High	Low	Low	Low	Low
16	Low	Low	High	Low	High	Low	High	Low	Low	Low	Low
17	Low	Low	High	Low	High	Low	High	Low	Low	Low	Low

### 3.4 Quality Assessment

**Figure 2:**
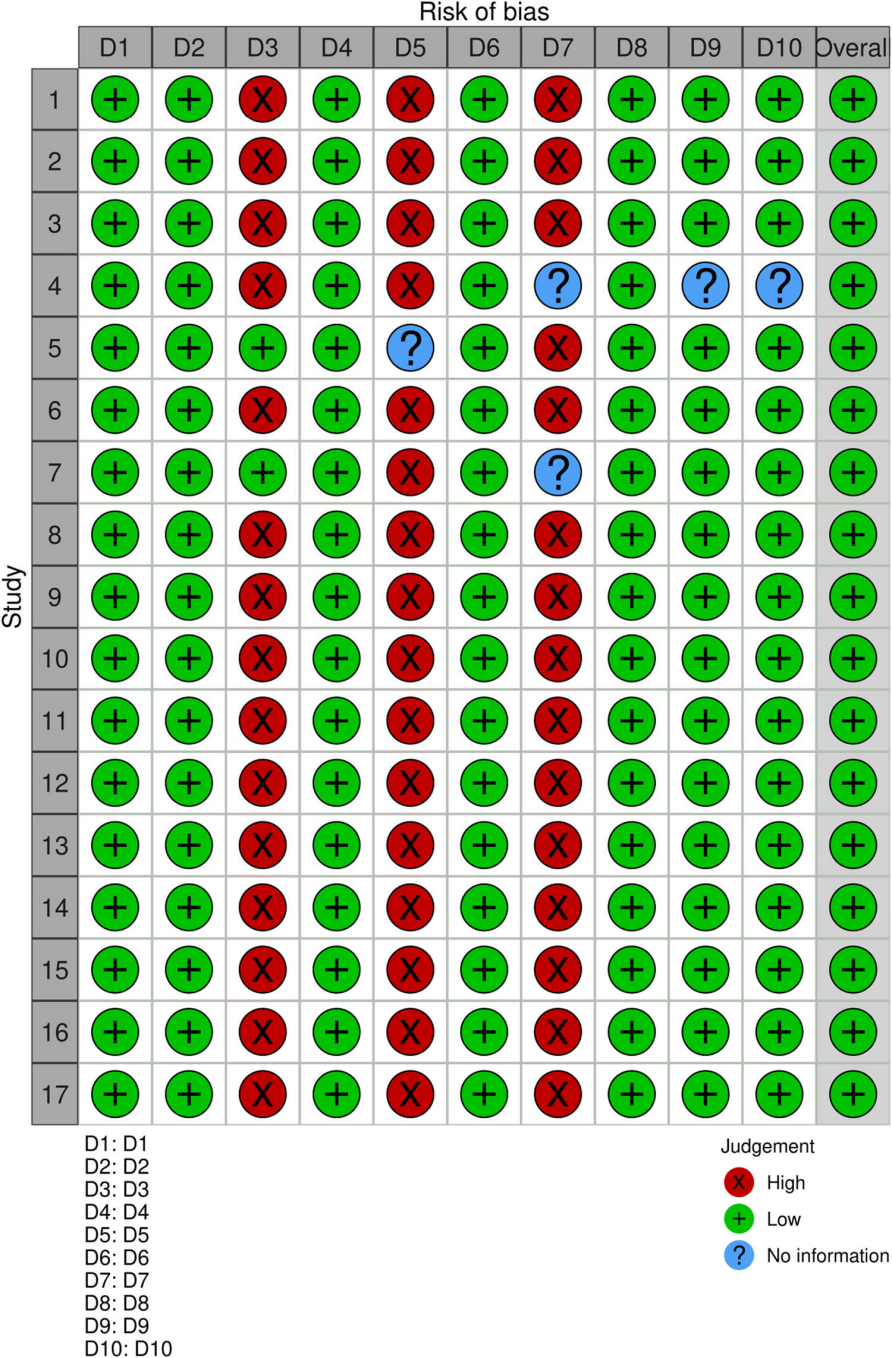
Quality assessment of the studies.

Although all studies were assessed as having an overall low risk of bias, certain domains (D3, D5, and D7) exhibited a high risk across most studies. This suggests that while the research may be generally reliable, specific areas of methodology may need careful consideration when interpreting the results. (Figure 2).

### 3.5 Meta‐Analysis

**Figure 3:**
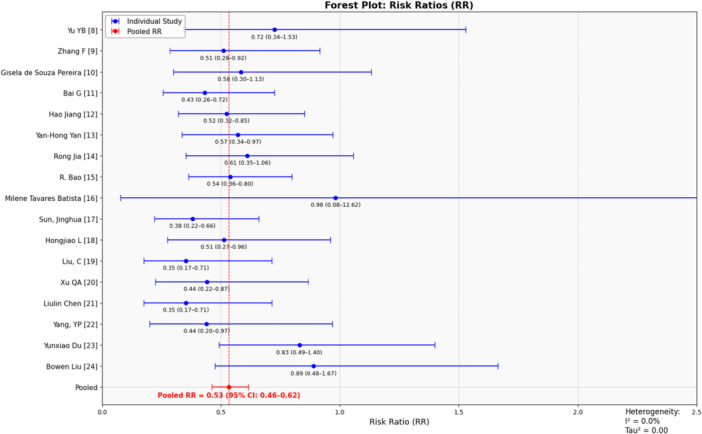
Forest plot: Risk ratios.

The forest plot illustrates the results of a meta‐analysis evaluating the effect of an intervention or condition on risk reduction, as measured by Risk Ratios (RR). The pooled RR, derived using a random‐effects model, is 0.53 (95% CI: 0.46–0.62), indicating a statistically significant 47% reduction in risk across studies. The CIs for individual studies, shown as horizontal lines, reveal varying levels of precision, with most studies reporting RRs below 1, suggesting a consistent reduction in risk. Heterogeneity metrics, including *I*² = 0.0% and Tau² = 0.00, indicate no significant variability between study results, implying that differences are primarily due to random error rather than systematic variation. The alignment of most individual RRs with the pooled estimate further reinforces the consistency and reliability of the findings. Overall, the graph provides strong evidence supporting the intervention′s effectiveness in reducing risk, with minimal heterogeneity across the included studies. (Figure 3).

## 4 Discussion

### 4.1 Overview of Main Findings

This systematic review highlights the potential of anticaries vaccines to reduce *Streptococcus mutans* colonization and dental caries development in preclinical models. Various platforms—including DNA‐based, nanoparticle‐adjuvanted, live‐vector, and passive immunization approaches—have demonstrated immunogenicity through both mucosal and systemic pathways. Antibodies such as secretory IgA (SIgA) and serum IgG play critical roles by neutralizing key virulence factors, preventing adhesion, and disrupting biofilm formation [25, 26].

### 4.2 Comparative Analysis of Vaccine Platforms

#### 4.2.1 DNA Vaccines

DNA‐based platforms, such as pGJAP and pGJA‐P/VAX, have demonstrated favorable immunogenicity. In a study it was found that intranasal administration of pGJAP produced significantly higher salivary SIgA levels than intramuscular injection, while serum IgG levels were higher in the intramuscular group, indicating route‐dependent immune responses [20]. Similarly, the pGJA‐P/VAX–bupivacaine complex significantly reduced caries scores in immunized rats, highlighting its prophylactic potential [19]. However, a consistent limitation across these DNA vaccine studies was low antigen expression.

To overcome this, Sun et al. (2010) developed a Salmonella‐based DNA vaccine using a dual‐promoter system, combining a prokaryotic nirB promoter with a eukaryotic promoter to enhance antigen expression under anaerobic conditions [17]. While immunogenicity improved, the use of live bacterial vectors raises safety concerns for human use, especially in immunocompromised individuals [28].

#### 4.2.2 MicroRNA Modulation

Li et al. addressed posttranscriptional suppression of antigen expression by identifying microRNA‐9 (miR‐9) as a key regulatory molecule that reduced DNA vaccine efficacy. Suppressing miR‐9 expression significantly increased antigen translation and SIgA production, offering a novel genetic enhancement strategy [18, 29].

#### 4.2.3 Nanoparticle‐Adjuvanted Vaccines

Nanoparticles are gaining momentum as next‐generation delivery platforms. Zhao et al. developed a ZIF‐8@PAc nanoparticle system that enabled pH‐responsive release, efficient antigen encapsulation, and activation of dendritic cells and central memory T cells. In their rat model, ZIF‐8@PAc significantly reduced *S. mutans* colonization and caries formation [8]. These findings suggest that metal–organic frameworks like ZIF‐8 may become a robust platform for mucosal vaccine delivery.

#### 4.2.4 Passive Immunization (IgY)

Liu B et al. investigated anti‐CatS‐IgY antibodies derived from chicken egg yolk. These targeted glucan‐binding protein B (GbpB) and glucosyltransferase B (GtfB), effectively inhibiting bacterial adhesion and biofilm formation. Their study demonstrated that IgY is a safe, stable, and specific tool for passive immunization, although likely requiring repeated administration for sustained protection [24].

### 4.3 Challenges in Vaccine Development

While promising, several limitations remain. DNA vaccines are limited by low antigen expression, which multiple groups have sought to overcome through promoter design [12]) and miRNA modulation [12, 14]. Mucosal delivery remains a challenge due to antigen degradation and the harsh oral environment. Nanoparticles such as ZIF‐8 offer promising solutions but remain in preclinical stages [8].

Animal model heterogeneity is another concern. Different species (mice, rats, hamsters) have varied immune responses and oral microbiota profiles, impacting generalizability. Inoculation methods, scoring systems, and follow‐up durations also differ widely across studies, contributing to methodological heterogeneity.

### 4.4 Promising Research Directions

Innovative platforms such as ZIF‐8@PAc and miRNA‐based vaccine enhancement represent a leap forward in immunogenicity and delivery efficiency [8, 14]. Studies demonstrated that optimizing the route of administration such as intranasal over intramuscular significantly enhances mucosal immunity [20, 27]. Collectively, these strategies signal a shift toward smarter vaccine designs tailored to oral pathogens.

Live‐attenuated mucosal vaccines, like the Cold‐Adapted Influenza Vaccine (CAIV), offer real‐world examples of success. Though not anticaries specific, CAIV′s safety and mucosal efficacy support the viability of similar platforms for caries prevention.

### 4.5 Strength and Limitations

Firstly, the meta‐analysis includes only one study per intervention arm, limiting the ability to draw strong comparative conclusions. Only studies published in the English language were included in this review, which may have introduced language bias and potentially excluded relevant data from non‐English sources. There are several methodological differences across the included studies, such as variations in vaccine type, dosage, route of administration, and follow‐up duration, all of which may have contributed to the observed heterogeneity in vaccine efficacy. A key limitation is that most of the available data are short‐term; therefore, it was not possible to assess or compare the long‐term effectiveness or duration of immunity induced by the different vaccine candidates. It also remains unclear which vaccines, if any, will provide sustained immune protection over time. Furthermore, booster dose requirements were often not addressed, despite their likely importance in maintaining long‐term immunity.A key methodological concern is that many studies used antibody levels as proxies for caries reduction, rather than directly measuring clinical caries outcomes. While immune responses are useful indicators, they may not fully predict protection against cariogenic lesions, especially in the absence of standardized immunological benchmarks.

Another significant limitation is the variability in animal models used across the studies. Differences in species can influence immune responses, vaccine pharmacokinetics, and oral microbiota, thereby affecting the outcomes and limiting the generalizability of the findings to humans. Additionally, differences in study environments, inoculation methods, and caries scoring systems further complicate cross‐study comparisons. The high heterogeneity detected in the meta‐analysis suggests that unmeasured or inconsistent variables likely influenced the effect sizes, underscoring the need for standardized protocols and more robust, long‐term, and human‐based clinical trials in future research.

### 4.6 Translational Implications and Future Directions

Translating these findings into human application depends on balancing efficacy, safety, and feasibility. Among the evaluated platforms, DNA vaccines (pGJAP, pGJA‐P/VAX) appear most clinically viable due to their scalability, safety, and dual immune stimulation. Nanoparticle platforms like ZIF‐8@PAc offer controlled delivery and superior immune activation but require further development and toxicity testing.

Passive immunization approaches such as anti‐CatS‐IgY offer rapid, short‐term protection and may serve as effective adjuncts for high‐risk populations or during acute disease outbreaks. While no anticaries vaccine has yet advanced to late‐stage clinical trials, early‐phase designs and proposals like mucosal subunit vaccines targeting PAc or Gtf antigens are under exploration.

Future studies should prioritize long‐term follow‐up, evaluate booster requirements, and transition toward human trials using standardized protocols and ethically sound frameworks. anticaries vaccines hold the potential to complement fluoride and mechanical plaque control, especially in underserved populations with limited dental care access.

## 5 Conclusions

In summary, there is excellent potential for dental caries vaccines to transform oral health in the future. Although there isn't currently a widely used vaccination, advances in the field and further study indicate that one could be developed in the near future to prevent dental caries. Various vaccines showed to have a prophylactic effect like Killed Formalin‐treated Donor strain 2 ‐ recombinant Protein Antigen c (KFD2‐rPAc), anti‐CAT‐SYIIgY (antibody) S. mutans on teeth and exhibit a persistent effect on reducing the colonization of S. mutans, both of which could contribute to the prophylactic effect against the development of caries A vaccination of this kind would significantly lower tooth decay prevalence and the financial and health costs that go along with it. Nevertheless, additional studies, clinical studies, and regulatory permissions are necessary to develop and successfully deploy. The search for dental caries vaccines is an appealing path for future developments in healthcare because of the possible influence on oral health worldwide.

However, to realize this potential, further progress is needed. Most existing evidence is derived from small‐scale or animal studies. Large‐scale human clinical trials are essential to assess safety, long‐term efficacy, optimal dosage, and delivery routes. Moreover, regulatory approval remains a significant hurdle, requiring robust data on manufacturing quality, risk‐benefit analysis, and post‐market surveillance plans. Securing consistent funding for such trials and overcoming ethical and logistical barriers in human testing are also critical. Ultimately, the path toward a dental caries vaccine will require coordinated scientific, regulatory, and financial efforts to bring a viable product to the global market.

In conclusion, anticaries vaccines offer promising immunogenic and protective responses across various platforms, particularly through mucosal delivery and nanoparticle adjuvants. However, these findings must be interpreted with caution due to key limitations in the available literature, including high heterogeneity, reliance on diverse animal models, and short‐term follow‐up. Future research should focus on standardized protocols, long‐term immunity, and early‐phase human trials to enable successful clinical translation.

## Author Contributions

Conceptualization: Gunjan Kumar and Mohmed Isaqali Karobari. Data curation: Gunjan Kumar, Payal Dash and Smruti Bhusan Nanda. Investigation: Gunjan Kumar, Payal Dash and Smruti Bhusan Nanda. Methodology: Gunjan Kumar, Smruti Bhusan Nanda and Mohmed Isaqali Karobari. Project administration: Gunjan Kumar and Mohmed Isaqali Karobari. Resources: Mohammad Fareed. Supervision: Mohmed Isaqali Karobari. Validation: Smruti Bhusan Nanda and Mohammad Fareed. Visualization: Mohammad Fareed. Writing – original draft: Gunjan Kumar, Payal Dash, and Mohmed Isaqali Karobari. Writing – review and editing: Smruti Bhusan Nanda, Mohammad Fareed, and Mohmed Isaqali Karobari. All the authors have read and approved the final manuscript.

## Ethics Statement

The authors have nothing to report.

## Consent

The authors have nothing to report.

## Conflicts of Interest

The authors declare no conflicts of interest.

## Supporting information

Supplementary file.

## Data Availability

All data were collected or calculated from the included published studies.
